# Supplementary material for: Analysis of Simian Endogenous Retrovirus (SERV) Full-Length Proviruses in Old World Monkey Genomes
Source: Genes (Basel). 2022 Jan 10;13(1):119. doi: 10.3390/genes13010119 (PMC8775094; doi:10.3390/genes13010119)
Supplement: Supplementary file 1 [file genes-13-00119-s001.zip › Table S1_Average nucleotide composition of Cer-SERV genomes.pdf]

Table S1. Average nucleotide composition (%) of Cer-SERV genomes.

Nucleotide	Complete genome	LTR	Gag-pro-pol	Env	SRV (type D retrovirus)[1]
A	31.4	22.6	33.3	29.7	33.3
T/U	26.0	27.2	25.7	27.5	25.8
C	23.9	33.2	21.9	24.3	22.8
G	18.7	17.0	19.1	18.5	18.2

1. Berkhout, B.; Grigoriev, A.; Bakker, M.; Lukashov, V.V. Codon and amino acid usage in retroviral genomes is consistent with virus-specific nucleotide pressure. *AIDS research and human retroviruses* **2002**, *18*, 133-141, doi:10.1089/08892220252779674.
