# Supplementary material for: Analysis of Simian Endogenous Retrovirus (SERV) Full-Length Proviruses in Old World Monkey Genomes
Source: Genes (Basel). 2022 Jan 10;13(1):119. doi: 10.3390/genes13010119 (PMC8775094; doi:10.3390/genes13010119)
Supplement: Supplementary file 1 [file genes-13-00119-s001.zip › Table S2_Estimate of the pattern of nucleotide substitutions in Cer-SERV-1 and Cer-SERV-2.pdf]

Table S2. Maximum Composite Likelihood estimate of the pattern of nucleotide substitutions in Cer-SERV-1 and Cer-SERV-2.

Cer-SERV-1 [1]	**A**	**T/U**	**C**	**G**
A	-	2.50	2.28	**15.25**
T/U	2.98	-	**19.29**	1.80
C	2.98	**21.15**	-	1.80
G	**25.22**	2.50	2.28	-

Cer-SERV-2 [2]	**A**	**T/U**	**C**	**G**
A	-	2.82	2.69	**14.54**
T/U	3.48	-	**18.95**	2.06
C	3.48	**19.88**	-	2.06
G	**24.53**	2.82	2.69	-

Each entry is the probability of substitution (*r*) from one base (row) to another base (column). Transitions are in shown in **bold**. Substitution pattern and rates were estimated under the Hasegawa-Kishino-Yano (1985) model (+G) [1]. A discrete Gamma distribution was used to model evolutionary rate differences among sites (5 categories, [+*G*], parameter = 0.2006). For estimating ML values, a tree topology was automatically computed. Relative values of instantaneous *r* should be considered when evaluating them. For simplicity, the sum of the *r* values is made equal to 100. All positions containing gaps and missing data were eliminated. Evolutionary analyses were conducted in MEGA6 [2]. There were a total of 8629 positions in the final dataset.

[1] The nucleotide frequencies are A = 31.17%, T/U = 26.14%, C = 23.84%, and G = 18.85%. The maximum Log likelihood for the ML tree was -40321.529. The analysis involved 55 nucleotide sequences.

[2] The nucleotide frequencies are A = 31.49%, T/U = 25.52%, C = 24.32%, and G = 18.67%. The maximum Log likelihood for the ML tree was -26259.835. The analysis involved 26 nucleotide sequences.

References

1. Hasegawa, M.; Kishino, H.; Yano, T. Dating of the human-ape splitting by a molecular clock of mitochondrial DNA. *Journal of molecular evolution* **1985**, *22*, 160-174, doi:10.1007/bf02101694.
2. Tamura, K.; Stecher, G.; Peterson, D.; Filipski, A.; Kumar, S. MEGA6: Molecular Evolutionary Genetics Analysis version 6.0. *Molecular biology and evolution* **2013**, *30*, 2725-2729, doi:10.1093/molbev/mst197.
