# Supplementary material for: Analysis of Simian Endogenous Retrovirus (SERV) Full-Length Proviruses in Old World Monkey Genomes
Source: Genes (Basel). 2022 Jan 10;13(1):119. doi: 10.3390/genes13010119 (PMC8775094; doi:10.3390/genes13010119)
Supplement: Supplementary file 1 [file genes-13-00119-s001.zip › Table S3_Characteristics of 81 Cer-SERV genomes.pdf]

Table S3. Characteristics of 81 full length Cer-SERV genomes

Sequence	Nt distance between LTRs	gag ORF, 1977 nt	prot ORF, 921 nt	pol ORF, 2616 nt	env ORF, 1713 nt	remarks	Cer-SERV genotype
NC_037673.1 Theropithecus gelada isolate Dixy chromosome 6, Tgel_1.0-A	0.029				stop at S419		SERV-1
NC_037679.1 Theropithecus gelada isolate Dixy chromosome 11, Tgel_1.0-B	0.013	1 nt del					SERV-1
NC_037687.1 Theropithecus gelada isolate Dixy chromosome 19, Tgel_1.0-A	0.002					4 ORFs open	SERV-1
NC_037687.1 Theropithecus gelada isolate Dixy chromosome 19, Tgel_1.0-B	0.006					4 ORFs open	SERV-1
NC_037679.1 Theropithecus gelada isolate Dixy chromosome 11, Tgel_1.0-A	0.029	stop at R290	1 nt del		7 + 2 nt del		SERV-1
NC_037672.1 Theropithecus gelada isolate Dixy chromosome 5, Tgel_1.0-A	0.004					4 ORFs open	SERV-1
NC_037685.1 Theropithecus gelada isolate Dixy chromosome 17, Tgel_1.0-A	0.015	stop at W180		1 nt ins, 4 nt del, 8 nt ins			SERV-1
NC_037681.1 Theropithecus gelada isolate Dixy chromosome 13, Tgel_1.0	0.022	4 nt del		2 nt del			SERV-1
NC_037671.1 Theropithecus gelada isolate Dixy chromosome 4, Tgel_1.0-A	0.033				1 nt ins		SERV-1
NC_037671.1 Theropithecus gelada isolate Dixy chromosome 4, Tgel_1.0-B	0.015			stop at L778	stop at S437		SERV-1
NC_037675.1 Theropithecus gelada isolate Dixy chromosome 7b, Tgel_1.0-A	0.013	stop at W359		stop at R622	2 + 5 nt ins		SERV-1
NC_037672.1 Theropithecus gelada isolate Dixy chromosome 5, Tgel_1.0-B	0.011	stops at S445, R502	TA-repeat (+ stops)	stop at W653			SERV-1
ref|NC_044982.1| Papio anubis isolate 15944 chromosome 7, Panubis1.0	0.020					4 ORFs open	SERV-1
NC_037676.1 Theropithecus gelada isolate Dixy chromosome 8, Tgel_1.0-A	0.008	1 nt ins	1 nt del	1 nt del			SERV-1
NC_018155.2 Papio anubis isolate 1X1155 chromosome 4, Panu_3.0-A	0.020	1 nt del		4 x 1 nt del, 2 x 1 nt ins, 2 x 2 nt ins, 2 x	1 nt del		SERV-1

				2 nt del, 1 x 7 nt del			
NC_018153.2 Papio anubis isolate 1X1155 chromosome 2, Panu_3.0-A	0.008	no startcodon (ATA)		1 nt del			SERV-1
NC_018157.2 Papio anubis isolate 1X1155 chromosome 6, Panu_3.0-A	0.015	1 nt del			2 nt del		SERV-1
NC_018155.2 Papio anubis isolate 1X1155 chromosome 4, Panu_3.0-B	0.019		1 + 2 nt ins		1 + 2 nt del		SERV-1
NC_041760.1:63495810-63503992 Macaca mulatta isolate AG07107 chromosome 7, Mmul_10	0.044				stop at R379		SERV-1
NC_027895.1 Macaca mulatta isolate 17573 chromosome 3, Mmul_8.0.1-A	0.008			1 nt del, 1 nt ins	2 x 1 nt del		SERV-1
NC_041761.1:47135738-47143910 Macaca mulatta isolate AG07107 chromosome 8, Mmul_10	0.015	1 nt del			1 nt ins, 2 nt del		SERV-1
NW_005093490.1 Macaca fascicularis unplaced genomic scaffold, Macaca_fascicularis_5.0 Scaffold38	0.037	1 nt del; 1 nt ins		stop at R650			SERV-1
NC_041766.1:21401809-21409977 Macaca mulatta isolate AG07107 chromosome 13, Mmul_10	0.033	1 nt del	1 nt del, 1 nt ins	1 nt del			SERV-1
Ensembl_provirus Macaca_mulatta_chromosome_Y 4120154 to 4128338 minus	0.026	stop at R290	10 nt del		2 nt del		SERV-1
NC_041754.1:103723616-103731779 Macaca mulatta isolate AG07107 chromosome 1, Mmul_10	0.035	1 nt ins		11 nt del, 4 x 1 nt del	1 + 1 nt del + 2 nt ins		SERV-1
BFBW01024194.1:15409-23817 Macaca fuscata fuscata DNA, contig24194, whole genome shotgun sequence	0.008	1 nt del			1 nt del		SERV-1
BFBW01009514.1:19393-27799 Macaca fuscata fuscata DNA, contig9514, whole genome shotgun sequence	0.006				2 nt del		SERV-1
BFBW01080171.1:1419-9817 Macaca fuscata fuscata DNA, contig80171, whole genome shotgun sequence	0.047	2 nt del		4 nt del	stop at Q416		SERV-1
BFBW01036568.1:13513-21917 Macaca fuscata fuscata DNA, contig36568, whole genome shotgun sequence	0.035	1 nt del (+3 nt del)	2 x 1 nt del	1 nt del			SERV-1

NC_037668.1 Theropithecus gelada isolate Dixy chromosome 1, Tgel_1.0-A	0.028			1 nt ins	1 nt ins		SERV-1
ref|NC_044976.1| Papio anubis isolate 15944 chromosome 1, Panubis1.0-B	0.050					4 ORFs open	SERV-1
NC_037673.1 Theropithecus gelada isolate Dixy chromosome 6, Tgel_1.0-B	0.008	1 nt ins					SERV-1
NC_037673.1 Theropithecus gelada isolate Dixy chromosome 6, Tgel_1.0-C	0.013	2 nt del	1 nt del	2 nt del			SERV-1
NC_037670.1 Theropithecus gelada isolate Dixy chromosome 3, Tgel_1.0-A	0.024	1 + 2 nt ins		1 nt del	1 nt del		SERV-1
NC_037678.1 Theropithecus gelada isolate Dixy chromosome 10, Tgel_1.0-A	0.013		stop at L271	2 x 1 nt del	10 nt del		SERV-1
NC_037687.1 Theropithecus gelada isolate Dixy chromosome 19, Tgel_1.0-C	0.033	1 nt del		stops at W35, L778			SERV-1
ref|NC_044976.1| Papio anubis isolate 15944 chromosome 1, Panubis1.0-A	0.050		stop at R249		2 x 1 nt del		SERV-1
NC_018154.2 Papio anubis isolate 1X1155 chromosome 3, Panu_3.0-A	0.033	1 nt ins	stop at W197	2 x 1 nt ins, 2 nt del	1 nt del		SERV-1
NC_018154.2 Papio anubis isolate 1X1155 chromosome 3, Panu_3.0-B	0.242	1 nt ins		4 x 1 nt del			SERV-1
NC_023658.1 Chlorocebus sabaeus isolate 1994-021 chromosome 17, Chlorocebus_sabeus 1.1-A	0.007			1 nt ins	1 nt ins, 1 nt del		SERV-1
NW_023666038.1:3456989-3465347 Chlorocebus sabaeus strain WHO RCB 10-87 unplaced genomic scaffold, Vero_WHO_p1.0 scaffold-6	0.000				stop at R31		SERV-1
NW_023666075.1:10876024-10884373 Chlorocebus sabaeus strain WHO RCB 10-87 unplaced genomic scaffold, Vero_WHO_p1.0 scaffold-43	0.007	stop at R22		4 nt del	1 nt ins, 1 nt del		SERV-1
NW_023666077.1:33850696-33859017 Chlorocebus sabaeus strain WHO RCB 10-87 unplaced genomic scaffold, Vero_WHO_p1.0 scaffold-45	0.034		stop at Q290	stops at L605, L778	1 nt del		SERV-1
ref|NC_044997.1| Papio anubis isolate 15944 chromosome Y, Panubis1.0	0.053			1 nt del	13 nt del		SERV-1
ref|NC_044989.1| Papio anubis isolate 15944 chromosome 14, Panubis1.0	0.029	stop at W56			1 nt del		SERV-1

ref|NC_044981.1| Papio anubis isolate 15944 chromosome 6, Panubis1.0-A	0.029		stop at R249		1 nt del		SERV-1
ref|NC_044981.1| Papio anubis isolate 15944 chromosome 6, Panubis1.0-B	0.033			stop at Q16	1 nt del		SERV-1
ref|NW_022164029.1| Papio anubis isolate 15944 unplaced genomic scaffold, Panubis1.0 scaffold387	0.036		stop at R249		1 nt del		SERV-1
ref|NC_044996.1| Papio anubis isolate 15944 chromosome X, Panubis1.0	0.015		stop at R249	stop at Q16	1 nt del		SERV-1
ref|NC_044981.1| Papio anubis isolate 15944 chromosome 6, Panubis1.0-C	0.024		stop at R249	stop at Q16	stop at Q547		SERV-1
ref|NW_022161492.1| Papio anubis isolate 15944 unplaced genomic scaffold, Panubis1.0 scaffold152	0.179			stop at Q16	2 x 1 nt del		SERV-1
Papio_anubis_chr_11_118991926-to-118992402-ensembl	0.028			2 x 1 nt ins	stop at Y222		SERV-1
Papio_anubis_chr_9_26805356-to-26810903-ensembl	0.028			2 x 1 nt ins	stop at Y222		SERV-1
gi|2246459|gb|U85505.1|STU85505 Simian endogenous retrovirus, complete genome	0.035			2 x 1 nt ins	stop at Y222		SERV-1
AB935214.1 Chlorocebus sabaeus DNA, simian endogenous retrovirus vero JCRB0111, complete sequence, cell_line: Vero JCRB0111	0.314					4 ORFs open	SERV-1
ref|NC_044995.1| Papio anubis isolate 15944 chromosome 20, Panubis1.0	0.034	3 x 1 nt del	stop at S150	stops at W24, Q83	2 x 1 nt del, 19 nt del		SERV-2
ref|NC_044983.1| Papio anubis isolate 15944 chromosome 8, Panubis1.0	0.032	2 x 1 nt del	stop at G102	stop at Y718	2 x 1 nt del		SERV-2
NC_023658.1 Chlorocebus sabaeus isolate 1994-021 chromosome 17, Chlorocebus_sabeus 1.1-B	0.031	1 nt del	stops at Q26, R249				SERV-2
NC_023649.1 Chlorocebus sabaeus isolate 1994-021 chromosome 8, Chlorocebus_sabeus 1.1	0.017		4 nt del				SERV-2
GPS_003667849.1 Chlorocebus sabaeus isolate 1994-021 unplaced genomic scaffold, Chlorocebus_sabeus 1.0 Scaffold369	0.049	1 nt ins	stop at R249	2 nt ins, 1 nt del	stops at K61, R541		SERV-2
NC_037685.1 Theropithecus gelada isolate Dixy chromosome 17, Tgel_1.0-C	0.072	1 + 2 nt ins; 1 nt del	1 nt ins (overlap gag)	1 nt ins, 1 nt del	4 nt del, 8 nt ins	shared provirus	SERV-2

NC_018168.2:46458393-46466808 Papio anubis isolate 1X1155 chromosome 17, Panu_3.0, whole genome shotgun sequence	0.084	1 + 2 nt ins; 1 nt del	1 nt ins (overlap gag)	1 nt del	4 nt del, 8 nt ins	shared provirus	SERV-2
NC_044990.1:45853202-45861607 Papio anubis isolate 15944 chromosome 15, Panubis1.0	0.075	1 + 2 nt ins; 1 nt del	1 nt ins (overlap gag)	1 nt ins, 1 nt del	4 nt del, 8 nt ins	shared provirus	SERV-2
PVJV010010813.1:8506-16907 Erythrocebus patas isolate BS28 EryPat_scaffold_21615, whole genome shotgun sequence	0.053	1 nt del	1 + 5 nt del	1 nt del, 11 nt ins	1 nt ins		SERV-2
NW_005093489.1 Macaca fascicularis unplaced genomic scaffold, Macaca_fascicularis_5.0 Scaffold37	0.045	1 nt del		stops at Q484, L778	1 nt del		SERV-2
NC_037668.1 Theropithecus gelada isolate Dixy chromosome 1, Tgel_1.0-D	0.036	stops at R22, E148, Q155		stop at L778			SERV-2
NW_018792497.1 Papio anubis isolate 1X1155 unplaced genomic scaffold, Panu_3.0 Scaffold31435	0.045	stops at L4, W588					SERV-2
NC_037668.1 Theropithecus gelada isolate Dixy chromosome 1, Tgel_1.0-C	0.033				no startcodon (ATT), stop at W500		SERV-2
NC_037676.1 Theropithecus gelada isolate Dixy chromosome 8, Tgel_1.0-B	0.011	1 nt ins	1 nt ins; 13 nt del	1 nt del	7 nt del		SERV-2
NC_037676.1 Theropithecus gelada isolate Dixy chromosome 8, Tgel_1.0-C	0.042	stop at W649	1 nt ins	1 nt del	stop at W237		SERV-2
NC_027894.1 Macaca mulatta isolate 17573 chromosome 2, Mmul_8.0.1-B	0.022		1 nt ins	2 x 1 nt del	1 nt ins		SERV-2
primary_assembly:Mmul_10:11:29816550:29825525	0.022	1 nt del			5 x 1 nt del		SERV-2
primary_assembly_Mmul_10_chr19_36701026-36710010	0.051	1 nt del	1 nt ins (overlap gag)	1 nt ins	1 nt ins		SERV-2
BFBW01007234.1:99392-107789 Macaca fuscata fuscata DNA, contig7234, whole genome shotgun sequence	0.026		1 nt ins		1 nt ins		SERV-2
BFBW01047892.1:22409-30792 Macaca fuscata fuscata DNA, contig47892, whole genome shotgun sequence	0.040	4 nt del		stop at Q83	1 nt ins		SERV-2

BFBW01059201.1:3610-11968 Macaca fuscata fuscata DNA, contig59201, whole genome shotgun sequence	0.068	1 nt del		16 + 8 nt del	1 nt del		SERV-2
NW_023666034.1:54525199-54533605 Chlorocebus sabaeus strain WHO RCB 10-87 unplaced genomic scaffold, Vero_WHO_p1.0 scaffold-2	0.015					4 ORFs open	SERV-2
NW_023666038.1:5204694-5213089 Chlorocebus sabaeus strain WHO RCB 10-87 unplaced genomic scaffold, Vero_WHO_p1.0 scaffold-6	0.015	1 nt del	stop at Q26	stop at L778			SERV-2
NW_023666038.1:1433607-1441999 Chlorocebus sabaeus strain WHO RCB 10-87 unplaced genomic scaffold, Vero_WHO_p1.0 scaffold-6	0.024		1 nt del	stops at W408, Q750	1 nt del		SERV-2
NW_023666038.1:220470-228861 Chlorocebus sabaeus strain WHO RCB 10-87 unplaced genomic scaffold, Vero_WHO_p1.0 scaffold-6	0.041	1 + 4 nt del		1 nt ins, 2 nt del	stop at S254		SERV-2
NW_023666033.1:79578207-79586623 Chlorocebus sabaeus strain WHO RCB 10-87 unplaced genomic scaffold, Vero_WHO_p1.0 scaffold-1	0.022	1 nt ins		11 nt ins			SERV-2
	mean nt distance LTRs SERV-1 (minus 3 'bad' seqs (in red)) = 0.023 ± 0.014	30/55 SERV-1 open	9/55 SERV-1 open	22/55 SERV-1 open	19/55 SERV-1 open		
	mean nt distance LTRs SERV-2 0.039 ± 0.019	Gag: 6/26 SERV-2 open	10/26 SERV-2 open	7/26 SERV-2 open	7/26 SERV-2 open		

All 4 ORFs are open in at least 6 SERV-1, and 1 SERV-2 provirus(es)
