# Supplementary material for: Analysis of Simian Endogenous Retrovirus (SERV) Full-Length Proviruses in Old World Monkey Genomes
Source: Genes (Basel). 2022 Jan 10;13(1):119. doi: 10.3390/genes13010119 (PMC8775094; doi:10.3390/genes13010119)
Supplement: Supplementary file 1 [file genes-13-00119-s001.zip › Table S4_Functional domains in encoded SERV proteins.pdf]

Table S4. Functional domains in encoded SERV proteins.

Gene	Domain or function	MPMV [a]	SERV-1 (% conservation)	SERV-2 (% conservation)	Reference(s)
Gag pp24/pp16	Late budding domain	PPPYX4PSAP	PPPYNKTXNEASAP (100%)	PPPYNGTXNGASA P (89%)	[1,2]
Gag Np24	Viral RNA packaging	KKPKR	NKPKK (97%)	NKQKK (67%)	[2]
Gag NC	Reverse transcription, RNA packaging, viral assembly	KNKEK	KEALK (100%)	KEALK (100%)	[3]
PR	N-terminal autoprocessing	SSDIY׀WVQPI	SSD/EIY׀WVQPI (80%)	SSDIY/WVQPI (33%)	[4]
PR/RT	Reverse transcription (G-patch domain)	QMLAQGYSPGK GLGKKENGILHPI PNQGQSNKKGF GNF	QMLTQGYTPGKGLG KGENGIPQPILVSGQ LDKKGFGNF (88%)	QMLTQGYTPGKGL GKKENGIPQPIPVS GQLDKKGFGNF (93%)	[5]
RT/RNAseH	Catalytic residues "D10, E48, D70, H124, D134"	D13, E53, D86, H131, D134	D10, E51, D85, H129, D132 (100%)	D10, E51, D85, H129 (100%), D132 (93%)	[6]
RT	Enzymatic activity (YXDD-box)	HYMDD	HYMDD (97%)	HYMDD (86%)	[7,8]
Env	Receptor-binding domain	SDGGGPQDKA R	SDGGGPLDI/TAR (90%)	SDGGGPLDTTR (86%)	[9]
Env	Immunosuppressive peptide	LQNRRGLDLLTA E QGGI	LQNRRGLDLLTAEQ/ KGGI (95%)	LQNRRGLDLLTAE QGGI (71%)	[10,11]
Env TM	Cytoplasmic domain, incorporation into and release of virions	IXXXYXXL	IXXXYXXL (100%)	IXXXYXXL (100%)	[12]

Conserved amino acids that have been shown to be essential for protein function are underlined

[a] MPMV, a variant of SRV-3, is the prototype D-type primate retrovirus, and has been the subject of detailed studies

References

1. Yasuda, J.; Hunter, E. A proline-rich motif (PPPY) in the Gag polyprotein of Mason-Pfizer monkey virus plays a maturation-independent role in virion release. *Journal of virology* **1998**, *72*, 4095-4103, doi:10.1128/jvi.72.5.4095-4103.1998.
2. Bohl, C.R.; Brown, S.M.; Weldon, R.A., Jr. The pp24 phosphoprotein of Mason-Pfizer monkey virus contributes to viral genome packaging. *Retrovirology* **2005**, *2*, 68, doi:10.1186/1742-4690-2-68.
3. Dostálková, A.; Kaufman, F.; Křížová, I.; Kultová, A.; Strohalmová, K.; Hadravová, R.; Ruml, T.; Rumlová, M. Mutations in the Basic Region of the Mason-Pfizer Monkey Virus Nucleocapsid Protein Affect Reverse Transcription, Genomic RNA Packaging, and the Virus Assembly Site. *Journal of virology* **2018**, *92*, doi:10.1128/jvi.00106-18.
4. Zábranský, A.; Andreánsky, M.; Hrusková-Heidingsfeldová, O.; Havlícek, V.; Hunter, E.; Ruml, T.; Pichová, I. Three active forms of aspartic proteinase from Mason-Pfizer monkey virus. *Virology* **1998**, *245*, 250-256, doi:10.1006/viro.1998.9173.
5. Křízová, I.; Hadravová, R.; Štokrová, J.; Günterová, J.; Doležal, M.; Ruml, T.; Rumlová, M.; Pichová, I. The G-patch domain of Mason-Pfizer monkey virus is a part of reverse transcriptase. *Journal of virology* **2012**, *86*, 1988-1998, doi:10.1128/jvi.06638-11.

6. Malik, H.S.; Eickbush, T.H. Phylogenetic analysis of ribonuclease H domains suggests a late, chimeric origin of LTR retrotransposable elements and retroviruses. *Genome research* **2001**, *11*, 1187-1197, doi:10.1101/gr.185101.
7. Johnson, M.S.; McClure, M.A.; Feng, D.F.; Gray, J.; Doolittle, R.F. Computer analysis of retroviral pol genes: assignment of enzymatic functions to specific sequences and homologies with nonviral enzymes. *Proceedings of the National Academy of Sciences of the United States of America* **1986**, *83*, 7648-7652, doi:10.1073/pnas.83.20.7648.
8. Argos, P. A sequence motif in many polymerases. *Nucleic acids research* **1988**, *16*, 9909-9916, doi:10.1093/nar/16.21.9909.
9. Sinha, A.; Johnson, W.E. Retroviruses of the RDR superinfection interference group: ancient origins and broad host distribution of a promiscuous Env gene. *Current opinion in virology* **2017**, *25*, 105-112, doi:10.1016/j.coviro.2017.07.020.
10. Sonigo, P.; Barker, C.; Hunter, E.; Wain-Hobson, S. Nucleotide sequence of Mason-Pfizer monkey virus: an immunosuppressive D-type retrovirus. *Cell* **1986**, *45*, 375-385, doi:10.1016/0092-8674(86)90323-5.
11. Lv, H.; Han, J.; Liu, J.; Zheng, J.; Zhong, D.; Liu, R. ISDTool: a computational model for predicting immunosuppressive domain of HERVs. *Comput Biol Chem* **2014**, *49*, 45-50, doi:10.1016/j.compbiolchem.2014.02.001.
12. Song, C.; Micoli, K.; Bauerova, H.; Pichova, I.; Hunter, E. Amino acid residues in the cytoplasmic domain of the Mason-Pfizer monkey virus glycoprotein critical for its incorporation into virions. *Journal of virology* **2005**, *79*, 11559-11568, doi:10.1128/jvi.79.18.11559-11568.2005.
