# Supplementary figures and images for: Analysis of Simian Endogenous Retrovirus (SERV) Full-Length Proviruses in Old World Monkey Genomes
Source: Genes (Basel). 2022 Jan 10;13(1):119. doi: 10.3390/genes13010119 (PMC8775094; doi:10.3390/genes13010119)

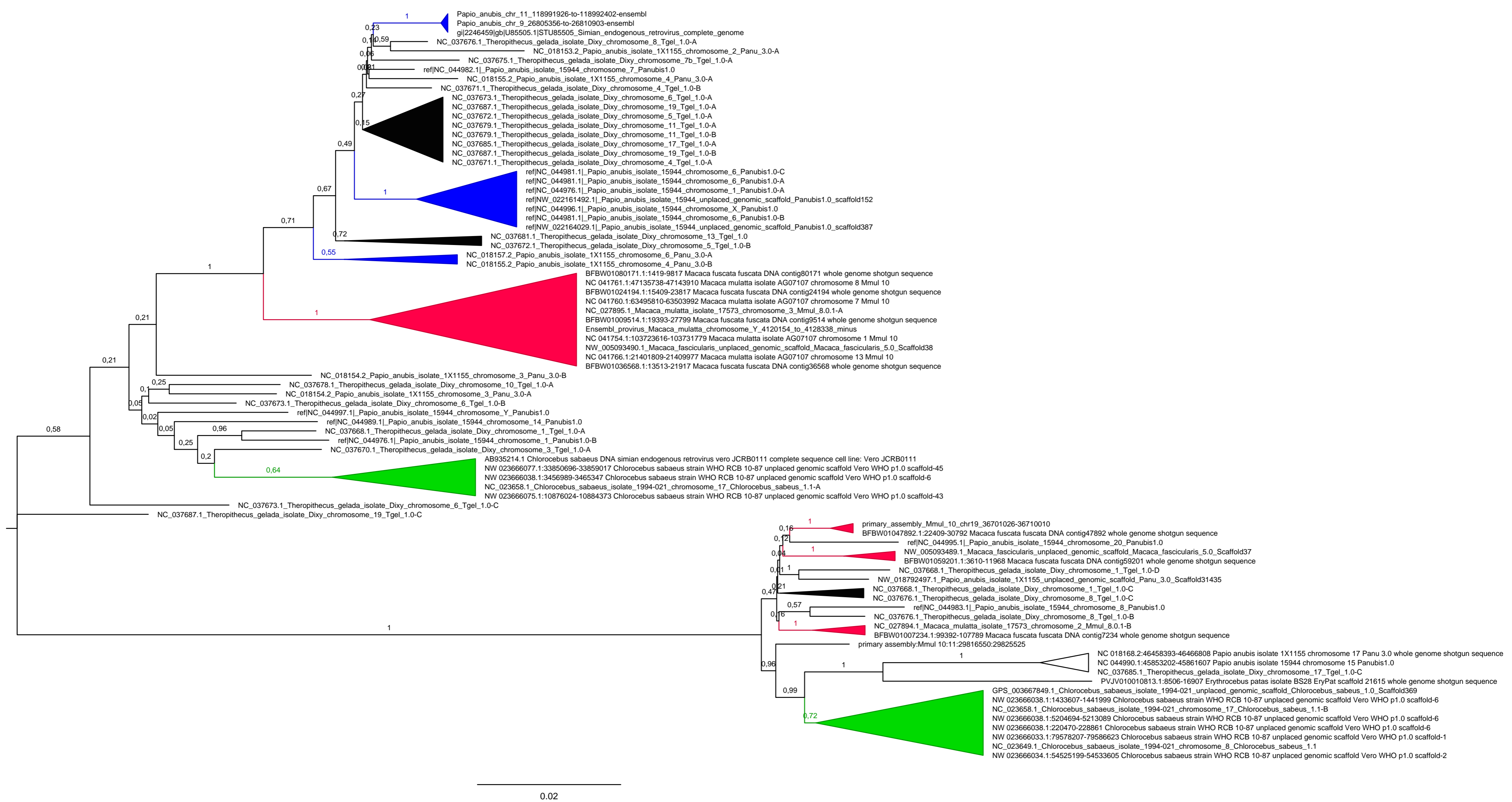

Supplement: Supplementary file 1 [file genes-13-00119-s001.zip › Figure S1.pdf]
